# Revision Anterior Cruciate Ligament Reconstructions at an Urban Tertiary Referral Center in Western India: A Retrospective Cross-Sectional Study

**DOI:** 10.7759/cureus.81654

**Published:** 2025-04-03

**Authors:** Dhruva Angachekar, Raunak Sharma, Shaswat Shetty, Shivam Patel, Abhay Narvekar, Sreedhar Archik, Sahil Lombar, Swastik Nakade, Megha Doiphode, Dhairya Angachekar

**Affiliations:** 1 Orthopedic Surgery, Paramount General Hospital and Intensive Coronary Care Unit (ICCU), Mumbai, IND; 2 Orthopedics, P.D. Hinduja Hospital and Research Centre, Mumbai, IND; 3 Orthopedics, Dr. KNS Memorial Institute of Medical Sciences, Barabanki, IND; 4 Sports Medicine, P.D. Hinduja Hospital and Research Centre, Mumbai, IND; 5 Orthopedics, Gleneagles Hospital, Mumbai, IND; 6 Otolaryngology, Dr. D. Y. Patil Medical College, Hospital and Research Centre, Pimpri Chinchwad, IND; 7 Medical Education, Paramount General Hospital and Intensive Coronary Care Unit (ICCU), Mumbai, IND

**Keywords:** anterior cruciate ligament (acl), anterior cruciate ligament (acl) tears, arthroscopic anterior cruciate ligament reconstructions, functional outcomes, return to sports, revision arthroscopic cruciate ligament reconstructions

## Abstract

Background: Anterior cruciate ligament reconstruction (ACLR) is a surgery performed to achieve knee stability and allow the knee to return to the preinjury level. There has been a significant increase in the number of primary arthroscopic ACLRs in recent years. However, the increase in primary surgeries has led to a proportional rise in revision ACLRs, primarily due to failure of the index surgery resulting from various factors.

Materials and methods: Hospital records from August 2020 to July 2023 were analyzed for revision ACLRs carried out at our center by a single surgeon with one year of postoperative follow-up. The patients who met the inclusion criteria and consented to participate in the study were called for clinical follow-up, and Lysholm scoring was performed to assess the functional outcome of the surgery.

Results: Twenty-eight patients met the inclusion criteria, 20 of whom consented to participate in the study. The mean age of the study population was 30.5 ± 6.82 years, with 85% (n=17) being males. There was a statistically significant difference in the Lysholm scores between primary and revision surgeries, with 50% (n=10) of patients having excellent outcomes (score >90) after index surgery and only 30% (n=6) falling into that category after revision surgery. There was a significant relationship between higher Lysholm scores and return to the preinjury level of play.

Conclusions: Revision arthroscopic ACLRs have good functional outcomes in the short and medium terms and are excellent for restoring knee stability. However, the outcomes are fair compared with those of primary arthroscopic ACLRs.

## Introduction

The anterior cruciate ligament (ACL) is among the most frequently injured ligaments of the knee. In the UK, there are approximately 30 ACL injuries per 100,000 people each year, and there are an estimated million ACL injuries worldwide [1]. The objectives of anterior cruciate ligament reconstruction (ACLR) are to allow patients to heal, stabilize the knee, and prevent additional injuries [2]. The frequency of revisions due to primary surgery failure is increasing in tandem with the number of primary ACLRs performed [3]. Revision reconstruction may be necessary for several reasons, including biological failure of the graft, trauma, undiagnosed secondary rotational instability, skeletal malalignment, varus/valgus instability, or graft failure owing to technical error of the surgeon [4].

The adult ACLR revision rate in the Danish registry is 4.1% after five years, whereas the revision rates in community registries in the US and Norway range from 0.9% to 1.5% [4,5]. Technical problems with revision ACLRs include retained hardware, bone tunnel defects, and improper tunnel locations, which call for specialized revision techniques. Furthermore, revision surgery still yields less satisfactory results than initial reconstructions do. Meniscal degeneration, chondral lesions, greater laxity, and a greater likelihood of graft failure are some of these worse outcomes [6,7].

Our study aimed to assess the demographic and injury trends of patients with re-ACL tears presenting at our institution between July 2020 and August 2023. Furthermore, we also analyzed the short- to medium-term functional outcomes of revision arthroscopic ACLRs and the factors affecting the return to sports after revision surgery.

## Materials and methods

**Figure 1:**
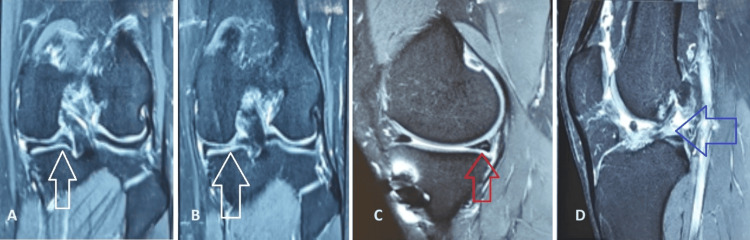
MRI images of the knee A and B: White arrow pointing toward the medial meniscus tear in the coronal section. C: Red arrow pointing toward the posterior horn of the medial meniscus tear in the sagittal section. D: Blue arrow pointing toward a full-thickness ACL graft rupture in the sagittal section. MRI: magnetic resonance imaging, ACL: anterior cruciate ligament

We reviewed the database of the hospital medical records department of our institution. The Institutional Ethics Committee of P.D. Hinduja Hospital and Research Center issued approval IEC-II(IRB)/1696/AL/24/17. The records were analyzed from August 2020 to July 2023, and all patients who met our inclusion criteria were contacted to participate in the study. The criteria for inclusion in the study were patients above 18 years of age who had a repeat ACL injury and who underwent revision arthroscopic ACLR using autografts at our institution by a single surgeon during the study period with a minimum of one year of postoperative follow-up. Patients who underwent single- and double-stage revision surgeries and surgeries with associated limb alignments were included in the study. Patients who did not fit the study duration and the defined age group were excluded from the study. Additionally, patients for whom allografts were used to reconstruct the ACL were excluded. Twenty-eight patients met the criteria, 20 of whom consented to participate in the study. The medical and radiological (Figure 1) records of these 20 patients were analyzed for data points related to the mechanism of primary injury and primary ACLR, as well as for repeat injury and revision surgery. They were called for follow-up to fill out the Lysholm scoring chart and underwent a clinical examination to analyze the surgical outcome after revision arthroscopic ACLR.

Statistical analysis was performed by entering the data in Microsoft Excel (Microsoft Corp., Redmond, WA, USA), and analysis was carried out in SPSS Statistics version 17.0 (IBM Corp., Released 2020. IBM SPSS Statistics for Windows, Version 27.0. Armonk, NY: IBM Corp.). Age categories, duration of return to sports in months, and Lysholm scores were compared with the Kruskal-Wallis test. Comparisons of Lysholm scores and operation times between revision and index surgeries and comparisons of tunnel sizes and durations of return to sports after physiotherapy across the revision and index surgeries were performed with paired t tests. Comparisons of Lysholm scores during revision surgery between those who returned and those who did not return to sports and comparisons of tunnel size during revision surgery and notchplasty were performed via the independent t-test. A p-value of <0.05 was considered to indicate statistical significance.

## Results

Among the 20 patients who participated in the study, 17 (85%) were male, whereas three (15%) were female. The patients were divided into four age groups: 20-25 years (n=4, 20%), 26-30 years (n=7, 35%), 31-35 years (n=5, 25%), and above 35 years (n=4, 20%). The mean age of our patients was 30.5 ± 6.81 years. Nine (45%) patients had left knee involvement, while the rest injured the right knee (n=11, 55%). Twenty-five percent (n=5) of the patients underwent treatment for primary ACL injury at our institution, while the remaining 75% (n=15) of the patients had undergone treatment at a different institution.

Most patients experienced their first ACL tear during sports activities (n=13, 65%). A total of 10% (n=2) of the patients were involved in a road traffic accident, whereas 10% (n=2) sustained injuries while dancing. Fifteen percent (n=3) of the patients sustained an ACL tear following a routine twisting injury. Fifteen percent (n=3) had associated medial meniscus injuries, whereas 40% (n=8) had lateral meniscus tears. One patient (5%) had an associated medial collateral ligament injury.

In 30% (n=6) of the patients, a six-stand semitendinosis with gracilis graft was used in the index surgery. In 55% (n=11) of the patients, a four-strand quadrupled semitendinosis graft was the graft of choice for the primary surgeon. For 5% (n=1) of the patients, a bone patellar tendon bone, peroneus longus, or central quadriceps tendon graft was used. Tibial fixation in the index surgery was performed with aperture fixation with a bioscrew in 40% (n=8) of the cases and suspensory fixation via a suture disc in 60% (n=12) of the cases. In 95% (n=19) of the patients, femoral fixation was suspensory in nature, whereas in 5% (n=1) of the patients, aperture fixation with a bone patellar tendon graft was performed on the femoral side. Meniscal repair was performed in 50% (n=10) of the patients, whereas 5% (n=1) of the patients underwent medial collateral ligament reconstruction along with ACLR. One patient each complained of persistent instability (5%) and a late surgical site infection (5%).

The mean duration between primary ACLR and revision ACLR was 56.15 months. Re-ACL tears occurred in 45% (n=9) of the patients while performing the same activity as during the initial ACL tear. Thirty percent (n=6) of the patients sustained injury while playing a different sport than during the index surgery. Fifteen percent (n=3) of the patients sustained a re-ACL tear due to a trivial twisting injury. In one patient (5%), there was an infection resulting in absorption of the graft, leading to failure, whereas one patient sustained injury in a road traffic accident.

After reviewing the history, medical and radiological records, operative images and videos, and intraoperative findings during revision surgery, we ascertained probable factors contributing to re-ACL tears. Forty percent (n=8) of the patients had improper tunnel placement, followed by 35% (n=7) of the patients who did not follow a proper physiotherapy protocol. 20% (n=4) of the patients experienced traumatic rupture of the graft, while 25% (n=5) of the patients returned early to competitive sports. A total of 10% (n=2) had lower limb alignment issues, which may have contributed to additional stress on the ACL graft. One patient (5%) experienced postinfection failure of the ACL graft, whereas 20% (n=4) had no technical cause of failure contributing to rerupture. In 45% (n=9) of the patients, multiple factors were noted that contributed to graft failure.

With re-ACL tears, 60% (n=12) of the patients had an associated medial meniscus injury, whereas 20% (n=4) of the patients had an associated lateral meniscus tear. On arthroscopic examination, 25% (n=5) of the patients had previous nonanatomic tibial tunnel placement, whereas 40% (n=8) of the patients had nonanatomic femoral tunnel placement. 10% (n=2) had both tibial and femoral tunnel widening.

In 90% (n=18) of the patients, the central quadriceps tendon graft was the graft of choice to reconstruct the ACL. A bone patellar tendon was used in one patient (5%), whereas in one patient (5%), a six-stand gracilis with a semitendinosis graft was used. Notchplasty was performed in 80% (n=16) of the patients, as larger-diameter grafts were taken for the revision procedures to prevent the graft from impinging on the notch roof. Ten percent (n=2) of the patients underwent associated medial meniscus subtotal meniscectomy, 5% (n=1) of the patients underwent partial medial meniscus meniscectomy, and 5% (n=1) of the patients underwent a medial and lateral meniscus balancing procedure. Five percent (n=1) of the patients underwent lateral meniscus repair, 5% (n=1) of the patients underwent medial and lateral meniscus repair, and 30% (n=6) of the patients underwent medial meniscus repair. One patient (5%) had an associated medial root tear, which was repaired. Lateral extraarticular tenodesis (LET) was performed in all patients. In 10% (n=2) of the patients, varus malalignment correction was performed with medial opening wedge high tibial osteotomy. Post-revision surgery, one patient (5%) experienced superficial surgical site infection, which resolved with local debridement and antibiotic treatment. One patient (5%) had undercorrection of varus deformity due to a break in the lateral bone hinge while performing the osteotomy. However, the patient was asymptomatic clinically.

**Figure 2:**
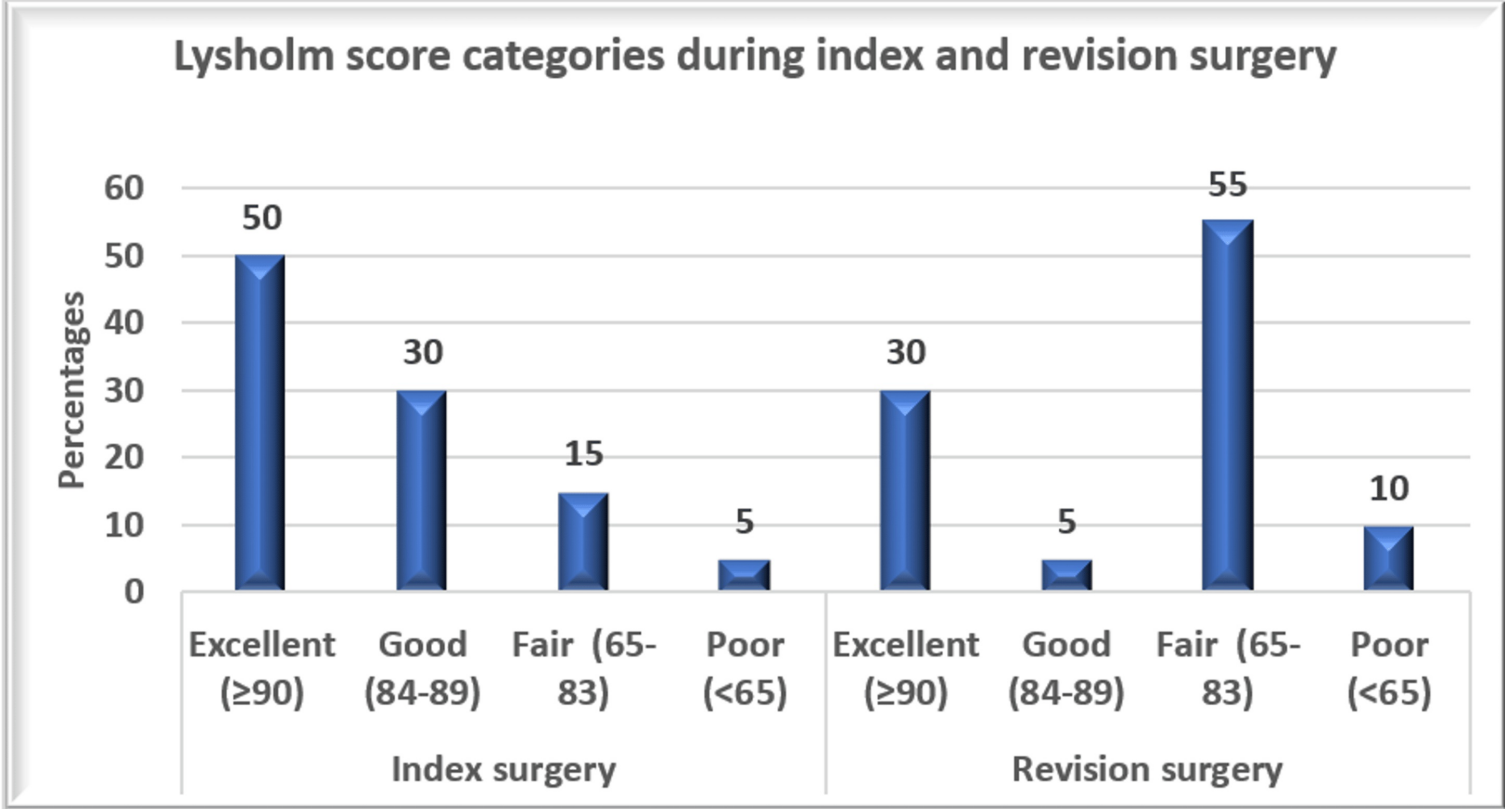
Lysholm score categories during index and revision surgery

The functional outcomes were tested according to the Lysholm scoring system, and the comparisons are given in Table 1 and Figure 2.

**Table 1:** Comparison of Lysholm knee scores and duration of postoperative rehabilitation across the different categories of age

		Revision surgery			Index surgery		
Categories	Total	Median	Interquartile range	p-value	Median	Interquartile range	p-value
Age categories and Lysholm knee scoring							
20-25	4	93	80.5-96	0.28	90	88-92.5	0.8
26-30	7	80	72-91		86	82-96	
31-35	5	80	75-86		86	84-90	
>35	4	73	69-78.5		91	75-96	
Age categories and duration of postoperative rehab (months)							
20-25	4	14	13 to 14	0.31	5.5	5 to 6	0.19
26-30	7	13	12 to 13		8	5 to 10	
31-35	5	12.5	12 to 13.5		8	7 to 8	
>35	4	12	12 to 12		7	6 to 8	

There was a statistically significant difference between increasing age and not being able to return to the same level of sports as before surgery (Table 2).

**Table 2:** Comparison of age group and return to sports

	Return to sports after revision surgery			
Age group (years)	No		Yes	
	n	%	n	%
20-25	1	25.0	3	75.0
26-30	1	14.3	6	75.7
31-35	3	60.0	2	40.0
>35	4	100.0	0	-
Total	9	100.0	11	100.0
Chi-square p-value = 0.03 (significant)				

The mean Lysholm score was notably lower in the revision surgery group (M = 79.65, SD = 10.5) than in the index surgery group (M = 87.6, SD = 7.5), with a mean difference of 8.0 points (95% CI: 3.2-12.7). The difference was statistically significant (p=0.002). Similarly, operation times were substantially longer in revision surgeries (M = 114.15 minutes, SD = 14.0) than in index surgeries (M = 65.05 minutes, SD = 14.9), with a mean difference of 49.1 minutes (95% CI: 39.1--59.1). This difference was highly significant (p≥0.001). There was a significant difference in the Lysholm score after revision surgery between patients who returned to sports and those who did not return to sports (Table 3).

**Table 3:** Comparison of Lysholm scores during revision surgery between those who returned and those who did not return to sports

Return to sports	Lysholm scores (revision surgery)			
	Mean	SD	Mean difference (95% CI)	p-value
No	72.3	8.1	13.3 (5.6-21.0)	0.002
Yes	85.6	8.3		

In revision surgeries, the mean tibial tunnel size was 10.5 mm (SD = 1.1), significantly larger than that in index surgeries, averaging 8.25 mm (SD = 0.8). The mean difference in tibial tunnel size between the two groups was 2.25 mm (95% CI: 0.97-2.7, p≤0.001). Similarly, for the femoral tunnel size, revision surgeries resulted in a larger mean size of 10.0 mm (SD = 1.1) than 8.0 mm (SD = 0.8) for the index surgeries. The mean difference in the femoral tunnel size was 2.0 mm (95% CI: 1.6-2.4, p≤0.001). There was a statistically significant relationship (p=0.001) between the graft diameter in patients who underwent notchplasty (M = 10.3 ± 1) and those who did not undergo notchplasty (M = 8.8 ± 0.5) along with revision ACLR.

## Discussion

The majority of the patients in our study group were males (n=17, 85%), with a mean age of 30.5 ± 6.8 years. Our study population's mean duration between primary and index surgeries was 56 months. These findings are similar to those of the study by Gallo et al., in which they retrospectively analyzed the surgical dates of 94208 patients who underwent revision ACLR surgeries from the California Office of Statewide Health Planning and Development Ambulatory Surgery Database. In their study, 63% of the participants were males; their mean age was 28 ± 12 years. The average time from primary ACLR to revision ACLR was 0.97 ± 0.55 years [8]. Similar results were observed in the study conducted by Buller et al., which examined trends in the surgical therapy of ACL injuries from 1990 to 2007 using data from the National Survey of Ambulatory Surgery and the National Hospital Discharge Survey databases. During the course of the study, more men than women underwent ACLR in both inpatient and outpatient settings, despite the gap gradually closing. The average patient age for outpatient procedures did not change between 1994 (28.8 years; 95% CI: 25.7-31.8) and 2006 (28.7 years; 95% CI: 26.6-30.8). Similarly, the mean patient age for inpatient ACLRs remained constant between 1990 (27.0 years; 95% CI: 24.7-29.4), 1996 (27.7 years; 95% CI: 24.0-31.4), and 2007 (26.0 years; 95% CI: 16.6-35.5) [3]. Most studies have shown a greater incidence of revision ACL surgeries in males than in females [4,9]. Our data revealed a greater percentage of males than did these studies, which may be attributed to the low number of participants in the study. Most studies have reported almost equal percentages of repeat ACL tears in both the right and left knees [10,11]. Our study had an almost equal distribution, with nine (45%) tears in the left knee and 11 (55%) in the right knee.

In Lind et al.’s study of the Danish registry for revision ACLRs, the most common cause of ACL injury was sports, with 80.8% of primary tears occurring during sporting activities. The most common sport in which injury was sustained was football, which is similar to our findings (n=9, 45%). This figure was 58.4% in the case of repeat ACL ruptures. Road accidents accounted for 7.9% of the primary tears and 14.9% of the revision tears. In our study, 10% (n=2) of the patients sustained injury from primary ACL tears due to RTAs, whereas 5% (n=1) of the patients sustained a repeat tear due to RTAs. Trivial injuries caused 15% (n=3) of primary and repeat ACL injuries in our study, which was 7% in the case of primary tears and 14.9% in the case of repeat tears in Linds’ study [4].

In some studies, technical failures in tunnel positioning account for more than 50% of ACL graft failures [11]. In our study, 40% (n=8) of the cases were associated with technical faults in tibial and femoral placements. In some studies, multiple causes of graft failure were noted in a single knee [12], which was similar to our study, where 35% (n=7) of the patients had multiple factors contributing to ACL graft failure.

The ideal choice of graft for ACLR is still unclear. This is especially the case in cases of revision ACL surgeries. The two main criteria determining graft selection are the surgeon's preference and the type of previous graft(s) utilized. However, a number of other factors also influence it, such as patient choice, the state of the contralateral knee, and tunnel dilatation [9]. Research has shown that a range of grafts can be used in revision ACLR surgeries [9,12-13]. These included autografts from the hamstring, patellar tendon, quadriceps tendon, peroneus longus, and a range of allografts. In our study, 90% (n=18) of the revision ACLRs were performed with a quadriceps tendon graft. Winkler et al., in their two-center retrospective study, concluded that quadriceps tendon grafts are a popular graft option for revision ACLRs with good clinical results [14]. Garofalo et al. reported a significant improvement in Lysholm scores and a significant reduction in tibial translation with KT-1000 testing during the postoperative period after performing a retrospective evaluation of 28 patients who underwent ACLR with quadriceps tendon autografts (with bone plugs). After following a nine-month rehabilitation plan, 94% of the patients had returned to their prior level of activity [15]. In a prospective study, Haner et al. evaluated 51 patients who underwent revision ACLR by hamstring or quadriceps tendon autografts. They reported no statistically significant differences in the KOOS subscores, Lysholm scores, or KT-1000 assessments at a minimum two-year follow-up [16]. After evaluating a small sample of 21 patients who underwent revision ACLR with quadriceps tendon autografts with bone blocks, Noyes et al. reported significant improvements in knee stability (by KT-1000 testing) and Cincinnati and IKDC (International Knee Documentation Committee) scores at a mean 49-month follow-up [12]. In 2022, Brinkman et al. conducted retrospective research on 58 revision ACLRs with more than two years of follow-up. They reported that all soft-tissue quadriceps tendon autografts had similar clinical results to BTB autografts. With the quadriceps tendon autograft, there may be a quicker return to sports and better early improvements in patient-reported outcomes that revert to normal after a year. Soft tissue quadriceps autografts should be considered a viable graft option for athletes undergoing revision ACL surgery [17].

In ACL surgery, there is considerable dispute regarding the indications and technique of anterolateral rotary stability (ALRI) (i.e., iliotibial band tenodesis versus anterolateral ligament restoration). ALRI is caused by anterolateral capsular injury, medial or lateral meniscus injury, and a steep tibial slope [18]. Alm et al., in their retrospective study of 75 patients with ACL graft failure conducted between 2016 and 2018, concluded that the addition of LET with revision ACLR resulted in significantly lower failure rates (5% vs. 21%, p=0.045) and a reduction in the frequency of a positive pivot shift in patients who underwent revision ACLR and high-grade anterior knee instability than in individuals who did not undergo LET surgery [19]. While LET plus revision ACLR did not increase the IKDC score, it did lower the failure rate and postoperative incidence of a positive pivot shift, according to retrospective multicenter research published by Trojani et al. on 163 patients with revision ACLR, of whom 51% underwent an additional LET procedure [20]. Additional LET was performed in all our patients (100%, n=20).

In the MARS study group, almost 74% of the patients had associated meniscal injury along with ACL graft ruptures [9]. In our study, 60% (n=12) of the revision cases involved associated medial meniscus tears, whereas 20% (n=4) of the cases involved lateral meniscus tears. Rotterud et al., in their cohort study of 8476 patients from Norway and Sweden, noted that almost 43% of the patients had associated meniscal injuries during primary ACL rupture [21]. Similarly, in our study, 50% (n=10) of the patients had an associated meniscal injury.

Notchplasty is a supplementary surgical treatment that involves enlarging and modifying the intercondylar femoral notch [22]. In their case-control study of 42 patients who underwent ACLR surgeries, Thompson et al. reported that graft rupture and revision surgery rates were lower in patients who underwent primary ACLR surgery with notchplasty [23]. However, Ranuccio et al., in their systematic review of the effects of notchplasty on ACLR surgeries, concluded that notchplasty may change the knee biomechanics, constrain the knee, and overload the graft, increasing the likelihood of graft failure or stiffness following surgery. They advised being cautious when evaluating the hazards associated with notchplasty during primary ACLR and saving it for revision surgery, notch osteophytosis, and surgical therapy for arthrofibrosis [24]. Considering the larger graft sizes and to avoid overconstraint of the knee joint with notch roof impingement, notchplasty was performed in approximately 80% (n=16) of our revision arthroscopic ACLRs.

In their meta-analysis comparing the functional and radiographic outcomes reported by patients and clinicians after revision versus primary arthroscopic ACLRs, Grassi et al. reported that, in comparison with patients who underwent primary ACLR, patients who underwent revision surgery had worse Lysholm Knee Scale scores, greater radiological evidence of tibiofemoral osteoarthritis, and worse surgeon-reported knee function. The meta-analysis included eight studies with a combined total of 300 revision ACLRs and 413 primary ACLRs; those who underwent revision surgery reported lower Lysholm Knee Scoring Scale scores, with a mean difference of 7.8 points [25]. These findings are similar to those of our study. Griffith et al. retrospectively reviewed 15 patients who underwent revision ACLR surgeries between 1998 and 2009 at their center. Their results revealed that before surgery, the average Lysholm score was 60; after surgery, it rose to 82 (p<0.001) [26]. This finding is similar to that of our study, where patients who could not return to similar activity levels after surgery had a mean Lysholm score of 72.3. In contrast, those who returned to similar levels of sports activities had a mean Lysholm score of 85.6. Gifstad et al., in their retrospective study comparing revision ACL surgeries with primary ACL surgeries, concluded that, compared with those of the primary group, the KOOS and Lysholm scores of the revision group were noticeably lower.

Additionally, patients in the revision group displayed more severe radiographic osteoarthritis, decreased muscle strength in the damaged knee, a greater decline in the Tegner activity score, and greater laxity, as assessed by the pivot shift test [27]. Most studies have shown that Lysholm scores are better in patients who undergo primary ACLRs than in those who undergo revision ACLRs.

Even in the adolescent age group, Saper et al. conducted a case series of revision ACLRs in athletes. They reported that only 68.4% of the athletes could return to pre-injury level sports [28]. In a systematic review by Glogovac et al. on return to sports after revision ACLR in athletes, 13 studies were analyzed. They reported that the percentage of return to sport at any level ranged from 56% to 100%, whereas the percentage of return to sport at the preinjury level ranged from 13% to 69%. The average period to return to sport ranged from 6.7 to 12 months [29]. In a meta-analysis of 31 studies involving 5365 patients, Andriolo et al. reported that although 73% of patients improved in terms of subjective or objective criteria, only 43% could resume their previous athletic activity level [30]. In our study, approximately 75% (n=15) of patients under 30 years of age returned to active sports following revision ACLR surgery; however, this percentage decreased to 40% (n=8) in the age group between 31 and 35 years. Beyond 35 years, none of the patients returned to active sports at the same level as before surgery.

The limitations of our study are that it is retrospective in nature with a chance of recall bias in the participants. It is a single-center and single-surgeon study with few participants and a short-term follow-up. With multiple concomitant injuries, confounding factors may bias the results. We recommend a multicenter, multi-surgeon study with a larger sample size and longer postoperative follow-up for further studies.

## Conclusions

Revision arthroscopic ACLR has good postoperative functional outcomes but is inferior to primary surgery. Younger patients have a greater chance of returning to sports after revision arthroscopic ACLR. The Lysholm scores and likelihood of returning to the same level of sporting activity decreased as the age of the patients increased, as younger patients are more motivated to return to sports in the medium term.
